# A Decalogue of Hygienic Maxims

**Published:** 1899-09

**Authors:** 


### A Decalogue of Hygienic Maxims.

Dr. Decornet, of Ferte-sur-Aube, has been proclaimed the winner of the prize which was offered by the publishing firm of Messrs. Hachette et Cie. in their "Annual Almanack" for 1897. The candidates, over 500 in number, were required to state briefly under ten heads the most effectual rules for preserving health, mental and bodily. The following are Dr. Decornet's ten maxims: 1. General Hygiene: Rise early, go to bed early, and in the meantime keep yourself occupied. 2. Respiratory Hygiene: Water and bread sustain life pure air and sunlight are indispensable for health. 3. Gastro-intestinal Hygiene: Frugality and sobriety are the best elixir for a long life. 4. Epidermal Hygiene: Cleanliness preserves from rust; the best kept machines last longest. 5. Sleep Hygiene: A sufficiency of rest repairs and strengthens; too much rest weakens and makes soft. 6. Clothes Hygiene: He is well clothed who keeps his body sufficiently warm, safeguarding it from all abrupt changes of temperature while at the same time maintaining perfect freedom of motion. 7. House Hygiene: A house that is clean and cheerful makes a happy home. 8. Moral Hygiene: The mind reposes and resumes its edge by means of relaxation and amusement, but excess opens the door to the passions and these attract the vices. 9. Intellectual Hygiene: Gaiety conduces to love of life, and love of life is the half of health; on the other hand, sadness and gloom help on old age. 10. Professional Hygiene: Is it your brain that feeds you? Don't allow your arms and your legs to become ankylosed. Dig for a livelihood, but don't omit to burnish your intellect and elevate your thoughts.

A VERY simple procedure will remove moles without having recourse to the knife, says a correspondent. Shave a match or sliver to as fine a point as possible, dip in carbolic acid and lightly touch the mole, care being taken to prevent the acid touching any other portion of the skin. Apply this every three or four days and the mole will gradually disappear, leaving space clean and healthy.

